# Brain-Targeted Polysorbate 80-Emulsified Donepezil Drug-Loaded Nanoparticles for Neuroprotection

**DOI:** 10.1186/s11671-021-03584-1

**Published:** 2021-08-18

**Authors:** Xiaojun Tao, Siyu Mao, Qiufang Zhang, Hongyuan Yu, Yu Li, Xiangling He, Shanyi Yang, Zhirong Zhang, Ziqi Yi, Yujiao Song, Xing Feng

**Affiliations:** 1 Key Laboratory of Study and Discovery of Small Targeted Molecules of Hunan Province and Department of Pharmacy, School of Medicine, Hunan Normal University, Changsha, 410013 China; 2 Hubei Key Laboratory of Wudang Local Chinese Medicine Research, Department of Pharmacology, Hubei University of Medicine, Shiyan, 442000 Hubei China; 3 Department of Hematology and Oncology of Children Medical Center, The First-Affiliated Hospital of Hunan Normal University, Changsha, 410005 China

**Keywords:** Pullulan polymer, Donepezil, Polysorbate 80, Alzheimer's disease, Brain targeting

## Abstract

Most Alzheimer’s disease drugs do not work efficiently because of the blood–brain barrier. Therefore, we designed a new nanopreparation (PS-DZP-CHP): cholesterol-modified pullulan (CHP) nanoparticle with polysorbate 80(PS) surface coverage, as donepezil (DZP) carrier to realize brain tissue delivery. By size analysis and isothermal titration calorimetry, we chose the optimal dosing ratio of the drug with nanomaterials (1:5) and designed a series of experiments to verify the efficacy of the nanoparticles. The results of in vitro release experiments showed that the nanoparticles can achieve continuous drug release within 72 h. The results of fluorescence observation in mice showed a good brain targeting of PS-DZP-CHP nanoparticles. Furthermore, the nanoparticle can enhance the drug in the brain tissue concentration in mice. DZP-CHP nanoparticles were used to pretreat nerve cells with Aβ protein damage. The concentration of lactate dehydrogenase was determined by MTT, rhodamine 123 and AO-EB staining, which proved that DZP-CHP nanoparticles had a protective effect on the neurotoxicity induced by Aβ_25–35_ and were superior to free donepezil. Microthermal perpetual motion meter test showed that PS-DZP-CHP nanoparticles have an affinity with apolipoprotein E, which may be vital for this nanoparticle targeting to brain tissue.

## Introduction

AD is a central nervous system disease with complicated pathological mechanisms that causes progressive cognitive dysfunction, but it is very hard to treat because of the difficulty in drug administration and the low concentration of drug that can reach brain tissue [1, 2]. Passage of the BBB has become a major difficulty in drug delivery system (DDS) research [3, 4]. Most drugs open the BBB through biological, chemical or physical means; among them, physical methods are now most commonly used clinically [5]. Due to the insuperable defects in intracranial drug delivery based on invasive technology, producing lipophilic prodrugs or active transport substrates through chemical modification of the drug surface has more advantages [6]. Among them, nanoparticles are one of the best choices to achieve intracranial drug delivery by actively targeting the BBB [7–9].

Nano-DDSs have become the focus of brain-targeting research and include polymer nanoparticles, organic nanoparticles, liposomes, nanofibers and micelles that have been designed to provide treatments and diagnostics [6, 10–12]. Both drug-loaded nanoparticles and small-molecule lipophilic drugs can pass through the BBB. The difference is that drug-loaded nanoparticles are more likely to pass through passive diffusion by adsorption on the capillary wall in the brain. In addition, free drugs face a second barrier, the blood-cerebrospinal fluid barrier (B-CSF) [13]. Unlike other barrier counterparts, most drugs are relatively permeable through the CSF and diffuse into the brain parenchyma. However, due to the very slow diffusion process, the concentration of free drugs in CSF is much higher than that in brain parenchyma, and the high concentration in the CSF will cause some toxicity [14, 15]. In this process, nanoparticles have unique and significant advantages. If the surface of the nanocarrier is coated with a hydrophilic surfactant, ApoE will adsorb to the surface, and CSF can promote the movement of nanoparticles toward the brain parenchyma through the space around blood vessels [16]. Nanoparticles coated with the nonionic surfactant polysorbate 80 prepared by Kreuter et al. [17] were the first drugs successfully delivered to the brain system and transported via adsorption of the serum protein ApoE in the plasma. Therefore, polysorbate 80 modification on the surface of nanoparticles to form a drug-specific composite allows targeting of drug and endogenous BBB receptor recognition, greatly increasing the bioavailability of drugs [18–20].

At present, the acetylcholinesterase (AChE) inhibitor donepezil (DZP) is commonly used for treatment of moderate AD, but due to its fat solubility, poor dissolution in *vivo*, and low oral bioavailability, conventional donepezil tablets must be taken daily to maintain the therapeutic effect [21, 22]. Because the cognitive ability of AD patients is severely impaired, which causes great inconvenience in maintaining a medication schedule, the development of a long-acting sustained-release DZP preparation is urgent. The amyloid cascade hypothesis suggests that the cause of AD is deposition of amyloid plaques around the brain parenchyma and cerebrovascular walls. A large number of diffuse spots are also observed in brain areas, which are composed of amorphous, highly fibrotic and insoluble extracellular Aβ deposits [23, 24]. Boridy et al. found that pullulan polysaccharide nanoparticles that are nontoxic and easily degradable can form a complex with the Aβ protein and effectively prevent protein aggregation and can be quickly cleared from cells to inhibit cell toxicity [25]. Cholesterol-hydrophobically modified pullulan (CHP) is an amphiphilic substance that can self-assemble into a nanostructure with a hydrophobic core and a sugar chain hydrophilic shell in an aqueous solution [26, 27]. At the same time, CHP nanoparticles can adsorb Aβ protein to prevent its deposition and aggregation, playing a synergistic role. As a nanocarrier, CHP has significant superiority.

Based on the above knowledge, the research team designed a DZP-CHP nanopreparation in the early stage and determined the optimal dosing ratio of the drug to the nanocarrier. Then, polysorbate was adsorbed on the surface of the nanoparticles to actively target passage through the BBB to achieve brain enrichment. In this study, a series of DZP-CHP nanosolutions were characterized, and the drug release process in vitro was explored and studied. After the nanoparticles were successfully prepared, Aβ25-35 was used to induce nerve cell damage to establish an AD cell model [28, 29]. Then, the protective effect of the DZP-CHP nanosolution was investigated in PC12 and SH-SY5Y cell models.

## Materials and Methods

### Materials

The following were used: cholesterol-hydrophobically modified pullulan (homemade) [30]; donepezil (Shanghai Ziqi Biotechnology Co., Ltd.); polysorbate 80 (Tianjin Fuchen Reagent Institute); indocyanine green (ICG) dye (Tianjin Baiying Biological Technology Co., Ltd.); polysorbate 80 (Tween 80, PS) (Tianjin Fuchen Reagent Office); black mice (Hunan Slake Jingda Laboratory Animal Co., Ltd.); Aβ25-35 (US Sigma); tetramethyl azozole salt (MTT) (US Sigma); newborn bovine serum (U.S. Gibco); lactate dehydrogenase kit (LDH) (Nanjing Jiancheng Biological Co., Ltd.); AO/EB double-staining fluorescence kit (Sino Pharmaceutical Group Chemical Reagent Co., Ltd.); and PC12 cells (rat adrenal pheochromocytoma cells) obtained from the Department of Neurology, Second Xiangya Hospital, Central South University. SH-SY5Y cells (human bone marrow neuroblastoma cells) were purchased from the ATCC Cell Bank (Manassas, VA, USA).

### Preparation of Nanoparticles

Three types of nanoparticles with different DZP and CHP ratios (w/w) (10:20, 4:20 and 2:20) were successfully prepared first via water dialysis according to methods reported in the literature [31]. A certain concentration of DZP-CHP nanoparticles was added to a beaker with a constant volume of 10 mL and then suctioned into another beaker containing polysorbate 80 (PS) emulsifier (concentration 0.7 mmol) to settle for 1 h. The mixture was then placed in an EP tube and sonicated for 3 min (output power 100 W, intermittent pulse working mode: pulse width 2.0 s, intermittent time 2.0 s). The operation was repeated three times until a uniform dispersion was obtained [32]. Polysorbate 80-emulsified donepezil drug-loaded nanoparticles (PS-DZP-CHP) were finally obtained after impurities were removed through filtration.

### Characterization of Nanoparticles

#### Nanoparticle Morphology

The shape, surface morphology and size of the DZP-CHP nanoparticles (DCPs) with DZP to CHP ratios of 1:2, 1:5 and 1:10 were analyzed with a Tecnai F20 transmission electron microscope. A drop of CHP, DZP-CHP and PS-DZP-CHP nanoparticles was placed on a carbon-coated copper mesh to form a thin liquid film. Then, 2% (w/v) phosphotungstic acid solution was used to obtain negative staining of the sample after natural drying of the film. The freshly prepared aqueous nanoparticle solution was added dropwise to a clean silicon wafer, dried at room temperature, and then placed under a JSM-6700F field emission scanning electron microscope to observe the surface structure.

#### Nanoparticle Size and Zeta Potential

The size, polydispersity coefficient (PDI) and zeta potential of DZP-CHP and PS-DZP-CHP nanoparticles were analyzed using dynamic light scattering (DLS). The average particle size and size distribution of the obtained homogeneous suspension were measured three times each.

### In Vitro Drug Release

The release of donepezil was measured using dynamic water dialysis. One milligram of DZP-CHP and PS-DZP-CHP nanoparticles was dissolved in 5 ml of phosphate-buffered saline (PBS, pH 7.4, concentration 0.01 M) and then transferred to a dialysis bag, which was kept in the same solution at a constant temperature of 37 °C with magnetic stirring. Four milliliters of PBS at 0, 0.5, 1, 2, 4, 8, 12, 24, 48 and 72 h was diluted with the same volume of PBS at the same pH. Ultraviolet–visible spectrophotometry was used to detect the absorbance of the dialysate at 312 nm at different times; the content of the solution was determined with a standard curve, and the release test was repeated three times in vitro. The release percentage of donepezil was calculated according to the following formula:

$$Q\% = {{(C{\text{n}} \times V + V{\text{n}}\sum\nolimits_{{{\text{t}} = {0}}}^{{\text{n}}} {{\text{Ci}}} )} \mathord{\left/ {\vphantom {{(C{\text{n}} \times V + V{\text{n}}\sum\nolimits_{{{\text{t}} = {0}}}^{{\text{n}}} {{\text{Ci}}} )} {(WNP \times LC\% }}} \right. \kern-\nulldelimiterspace} {(WNP \times LC\% }})$$

Cn is the sample concentration at the Tn time point, μg/mL; V is the total volume of PBS release solution, mL; Vn is the PBS release liquid volume at the Ti time point, mL; and Ci is the donepezil concentration at the Ti time point, μg/mL.

### Isothermal Titration Calorimetry (ITC)

A certain concentration of PS solution was dripped onto CHP nanoparticle solutions, and the changes in heat were measured with ITC (vip-itc, Microcal, Northampton, MA, USA). The CHP nanoparticle solution included three types of nanoparticles with different DZP-to-CHP ratios (1:2, 1:5 and 1:10). All solutions were degassed before titration. The temperature of the entire system was kept constant at 25 °C.

### Animal Experiments to Observe Brain Targeting

#### Preparation of ICG-Labeled Donepezil CHP Nanoparticles

Four hundred milligrams of CHP-DZP and 20 mg of ICG were weighed using an analytical balance, and an appropriate amount of DMSO was added for mixing and dissolving thoroughly. Then, the solution obtained above was added dropwise to the dialysis bag with a pipette, and the distilled water was replaced once every hour. Three hours later, the distilled water was replaced every 2 h, and 400–800 mL of distilled water was added each time for 48 h until the DMSO was dialyzed completely. After that, the above solution was transferred with a pipette into a volumetric flask to obtain a constant volume and then treated with an ultrasound wave for 2 min. Filtration through a 0.45-μm filter membrane resulted in ICG-labeled DZP-CHP nanoparticles (ICG-DZP-CHP), which were packed separately and stored in a refrigerator at 4 °C for future use.

#### Preparation of Emulsified Fluorescent Donepezil CHP Nanoparticles

An appropriate amount of ICG-DZP-CHP was placed into a 10-mL beaker, and 1 ‰ (v/v) polysorbate 80 (PS) emulsifier was added. The beaker was kept for 1 h and then transferred into an EP tube for ultrasonication for 2 min at 100 W. The above operation was repeated three times until a uniform nanosolution was obtained. Finally, filtered and ICG-labeled emulsified donepezil drug-loaded nanoparticles were obtained (PS-ICG-DZP-CHP).

#### MST Experiments to Verify Binding of the APOE to Nanoparticles

All MST experiments were performed on a Monolith NT.115 system (201810-BR-N024). All solutions were prepared with deionized water and analytical-grade reagents. Buffers were prepared and stored at room temperature. Protein samples were kept on ice until use [33]. PS-ICG-CHP nanoparticles (55.6 μM) were diluted to 40 nM with deionized water, and ICG was loaded for fluorescence. APOE solution (30 μl, 55.6 μM) was prepared, and 16 capillary tubes were labeled 1 to 16; first, 20 μl of APOE was added to tube 1, and 10 μl was added to tubes 2 to 16. Then, 10 μL of solution was transferred from tube 1 to tube 2 and mixed thoroughly. After that, 10 μl of solution was removed from tube 2 and transferred to tube 3. This operation was repeated until 10 μL of solution was finally removed from tube 16 to ensure that the solution in each tube was of the same volume. Ten microliters of diluted nanoparticles was added to each tube and mixed thoroughly to start the measurement. The MST test data were analyzed with NT-analysis software, and KD fitting was performed according to the law of mass action following the software instructions.

#### In Vivo Fluorescence Imaging Technology for Brain Targeting Observation

A batch of healthy black mice weighing approximately 18–22 g each were chosen and randomly divided into 2 groups: the PS-ICG-DZP-CHP group and the ICG-DZP-CHP group. Every mouse was injected with 200 μl of 200 μg/ml of the above group drugs via the tail vein, and 0.5 h later, the mice were anesthetized with 1% pentobarbital sodium (50 mg/kg). After that, all the mice were placed in the photographing area of the live imager with the imaging parameters set to an excitation wavelength of 765 nm-815 nm and an absorption wavelength of 815 nm-845 nm to obtain a fluorescence image of the whole animal. After imaging, all the mice were dissected, and the kidney, heart, spleen, lung, liver, and brain were removed to obtain fluorescence images. The imaging parameters were consistent with those described above.

### Study on Tissue Distribution of Nanoparticles

#### Grouping and Sampling of Mice

Forty-five C57BL/6 mice were randomly divided into 15 groups: 5 groups were injected with free donepezil (free group), 5 with donepezil nanoparticles (nano-group), and another 5 with PS-modified donepezil nanoparticles (PS group) at 0.25 mg/kg through the vein tail. Then, blood was taken at 1 h, 3 h and 6 h after injection. After that, all the animals were sacrificed, and the heart, brain, liver, and kidney tissues were collected and shredded. Then, 0.2 g of the above tissue was weighed precisely and added to approximately 1 ml of 0.9% NaCl solution and homogenized with a homogenizer (65 Hz, 150 s). One hundred microliters of tissue homogenate was accurately drawn into a 1.5-mL EP tube with 0.7 mL of methanol, vortexed to mix for 30 s for protein precipitation, and then centrifuged at 12,000 r·min^−1^ for 10 min. Finally, 100 μL of supernatant was transferred into an injection bottle for analysis.

#### Determination Method

HPLC was first used for examination, but the sensitivity was not sufficiently high. Therefore, follow-up LC–MS experiments were performed, which showed strong specificity and no endogenous substances interfering with the drug determination. The LC–MS protocol complied with the guidelines for the determination of biological samples. The chromatographic conditions were as follows: mobile phase A, water (containing 0.1% formic acid); mobile phase B, methanol (containing 0.1% formic acid); isocratic elution: A30%-B70%; flow rate, 0.3 mL· min^−1^; column temperature, 35 °C; injection volume, 10 μL. The collision conditions were as follows: electrospray ionization source (ESI) temperature, 150 °C; desolation gas flow rate, 550 L·h^−1^; desolation gas temperature, 500 °C. The conditions for positive ion detection were as follows: capillary voltage, 3 kV; cone voltage, 30 V; scanning mode, multiple reaction monitoring (MRM).

### Cell Experiments

#### Cell Culture and Passage

PC12 and SH-SY5Y cells were cultured in high-sugar DMEM supplemented with 10% (v/v) heat-inactivated fetal bovine serum (FBS) and 1% (v/v) penicillin and streptomycin and then kept in an incubator containing 5% CO_2_ at 37 °C. Cells were used in various experiments or passaged as soon as they reached 80% confluence. Before experiments, PC12 and SH-SY5Y cells were seeded on collagen type I precoated plates at the required cell density according to the experimental scale.

#### Cell Cryopreservation

When observed to grow to log phase under a microscope, PC12 and SH-SY5Y cells were frozen and washed twice with PBS, trypsinized to form a cell suspension and placed in a sterile centrifuge tube for centrifugation collection (1000 r × min^−1^, 3 min). Then, cell cryopreservation solution was added, and the cells were kept in a tube with the cell name and date marked. The cells were placed in a refrigerator at 4 °C for 1 h, − 20 °C for 2 h, − 80 °C (frozen) overnight and finally transferred to a liquid nitrogen tank.

#### MTT Method for Detecting Cell Survival Rate

Cell viability was measured using an MTT reduction assay. Briefly, PC12 and SH-SY5Y cells were seeded in 96-well plates (precoated with type I collagen) at a density of 1 × 10^4^ cells/mL to allow cells to adhere to each well. After incubation for 24 h, the cells were preincubated with different concentrations of donepezil CHP nanosolution or free donepezil solution for 2 h. Subsequently, Aβ25-35 (final concentration 20 μM) was added to each well. The treated 96-well plate was incubated at 37 °C for 24 h. After that, MTT (50 μL, 5 mg/mL) was added and incubated with the treated cells at 37 °C for 4 h. Finally, the medium was carefully removed, and the formazan crystals were dissolved in 150 μL of DMSO. The absorbance was obtained at 490 nm using a microplate reader. Cell viability is expressed as the percentage of living cells in the treated group and the percentage of living cells in the untreated control group.

#### Determination of LDH Activity in Cell Supernatant

PC12 and SH-SY5Y cells were seeded into 96-well culture plates (precoated with type I collagen) at densities of 2 × 10^5^ and 3 × 10^5^ cells/mL, respectively. After incubation for 24 h, the cells were preincubated with different concentrations of donepezil CHP nanosolution or free donepezil solution for 2 h. Subsequently, Aβ25-35 (final concentration 20 μM) was added to each well. LDH activity was measured according to the instructions provided by the kit. Briefly, the cultured cells with medium were collected and then centrifuged at 3500 rpm. The supernatant (50 μL) was mixed with an equal volume of reactant to initiate the LDH reaction. The absorbance was obtained at 450 nm using a microplate reader, and LDH activity was calculated.

#### AO/EB Staining Method to Observe Apoptosis Morphology

AO/EB fluorescent dye was used to evaluate the characteristics of apoptotic cells. PC12 and SH-SY5Y cells were seeded in black 12-well culture plates (precoated with type I collagen) at densities of 3 × 10^5^ and 4 × 10^5^ cells/well, respectively. After incubation for 24 h, the cells were preincubated with different concentrations of donepezil CHP nanosolution or free donepezil solution for 2 h. Subsequently, Aβ25-35 (final concentration 20 μM) was added to each well. After the treatment, operations were performed according to the kit. Light was avoided throughout the experiment. Finally, cell morphology was observed.

#### Rhodamine 123 Staining Method for Detection of Mitochondrial Membrane Potential

MMP was measured using rhodamine 123 (Rh123) fluorescent dye, a cell-permeable cationic dye that is preferentially distributed into mitochondria due to its highly negative properties. PC12 and SH-SY5Y cells were seeded in black 24-well culture plates (precoated with type I collagen) at densities of 2 × 10^5^ and 3 × 10^5^ cells/well, respectively. After incubation for 24 h, the cells were preincubated with different concentrations of donepezil CHP nanosolution or free donepezil solution for 2 h. Subsequently, Aβ25-35 (final concentration 20 μM) was added to each well. After the treatment, the cells were washed with PBS and incubated with 10 μg/mL rhodamine 123 for 30 min in the dark at 37 °C. After incubation, the cells were washed 3 times with PBS, and the fluorescence intensity was measured at 488 nm and 510 nm using a fluorescence plate reader.

#### Statistical Processing and Data Analysis

All the experiments were repeated three times, and the results are expressed as the mean ± standard deviation. GraphPad Prism statistical software was used, and one-way ANOVA, Student's *t *test and other methods were used for statistical analysis. *P* < 0.05 indicates that a difference is statistically significant.

## Results

### Characteristics of Nanoparticles

**Fig. 1:**
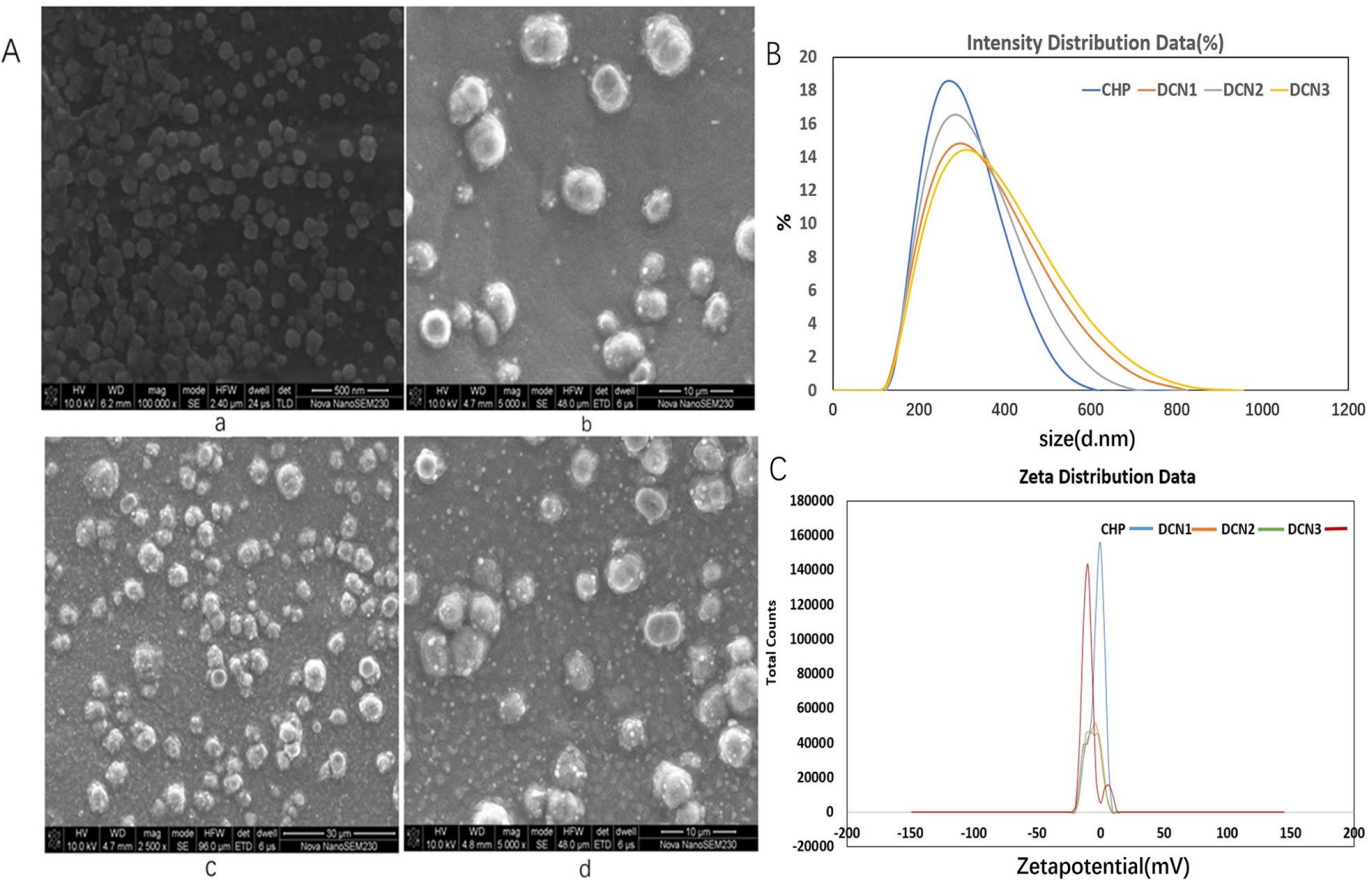
Scanning electron microscopy images (**a**, a-CHP, b-DCN1, c-DCN2, d-DCN3), size distribution (**b** a-CHP, b-DCN1, c-DCN2, d-DCN3) and zeta potential (**c** a-CHP, b-DCN1, c-DCN2, d-DCN3) of nanoparticles

CHP self-aggregates to form nanoparticles with hydrophobic cores, which can be loaded with DZP. DZP-loaded CHP nanoparticles (DCNs) with drug to nanomaterial ratios of 1:2, 1:5 and 1:10 were named DCN1, DCN2 and DCN3. According to scanning electron microscopy results, CHP nanoparticles showed a spherical structure, and after DZP loading, the DCNs were also spherical, as shown in Fig. 1. The mean size and zeta potential of the CHP nanoparticles were 257 ± 3.05 nm and − 2.81 ± 0.27 mV, respectively. After DZP loading, the mean sizes were 273 ± 3.72, 260.7 ± 1.76 and 266.8 ± 4.56 nm, and the zeta potentials were -6.20 ± 0.40, − 5.75 ± 0.64 and -9.30 ± 0.39 mV for DCN1, DCN2, and DCN3, respectively, as shown in Table 1. The percentages of drug entrapment were 42.00 ± 5.65%, 86.54 ± 1.31% and 59.71 ± 4.43%, and the percentages of drug loading were 12.02 ± 1.90%, 13.42 ± 2.03% and 7.40 ± 1.72%, respectively.

### ITC Measurement

**Fig. 2:**
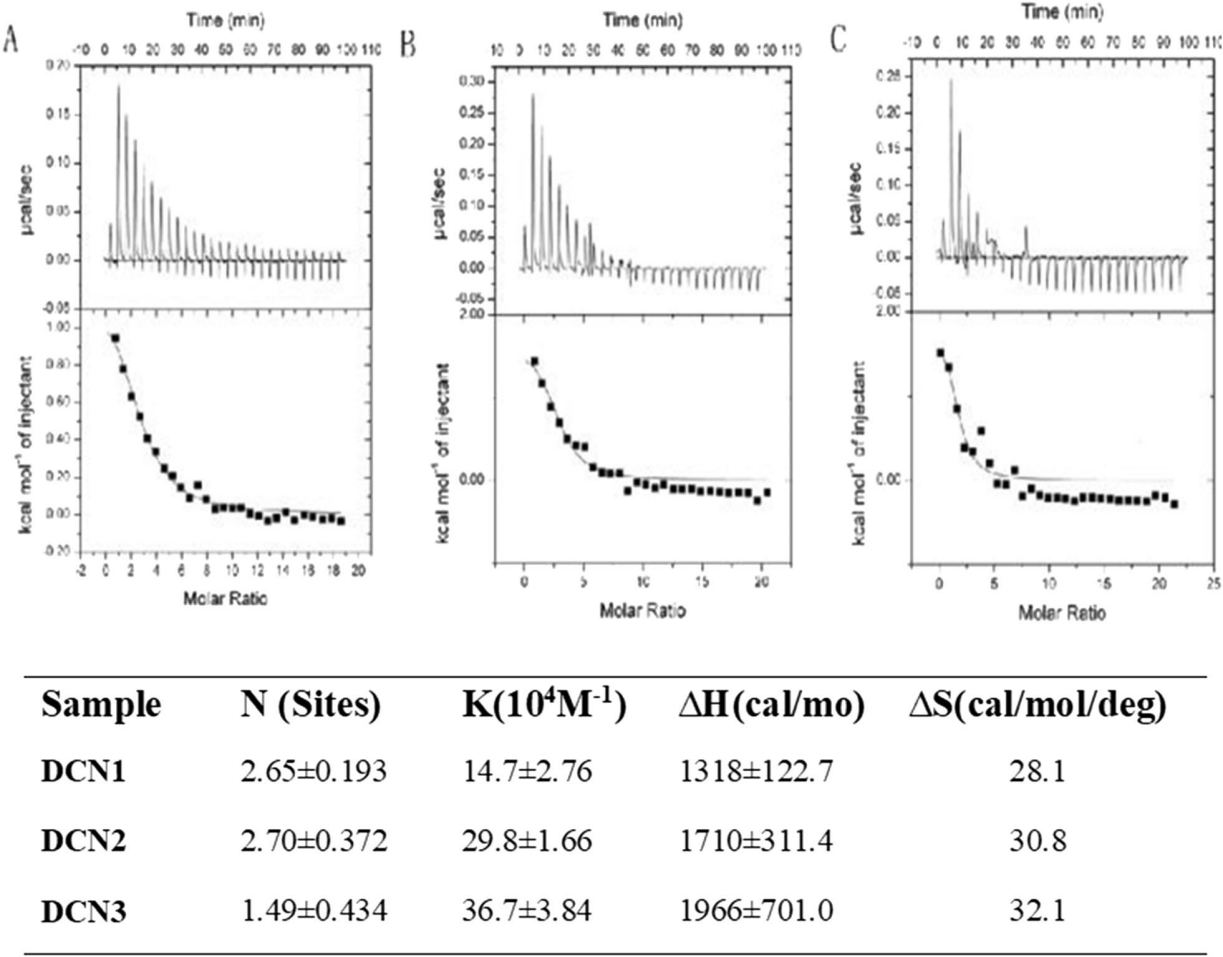
Isothermal calorimetry data for PS (0.9 mM) titration into **a** DCN1, **b** DCN2 and **c** DCN3 (0.02 mM) solutions at 25 °C. Degree of coverage, affinity (KA) and enthalpy and entropy changes for PS binding with nanoparticles (NPs) after titration into NP solutions

For DCNs, during the entire reaction process, the reaction mainly showed an upward peak (Fig. 2), and the reaction was endothermic because the upward peak indicates a heat-releasing reaction. Therefore, PS can spontaneously be adsorbed on the DCN surface. The PS affinity was (14.7 ± 2.76) × 10^4^ M^−1^, (29.8 ± 1.66) × 10^4^ M^−1^ and (36.7 ± 3.84) × 10^4^ M^−1^, and the degree of PS coverage was 2.65 ± 0.193, 2.70 ± 0.372 and 1.49 ± 0.434 for DCN1, DCN2 and DCN3, respectively. This result indicates that PS adsorbed to the DCN surface with a high affinity and had a greater coverage amount on DCN2. *∆H* > 0 and *∆S* > 0 indicates that the three particles were primarily bound through hydrophobic interactions with PS.

### Characterization of the Three Nanoparticle Types

**Fig. 3:**
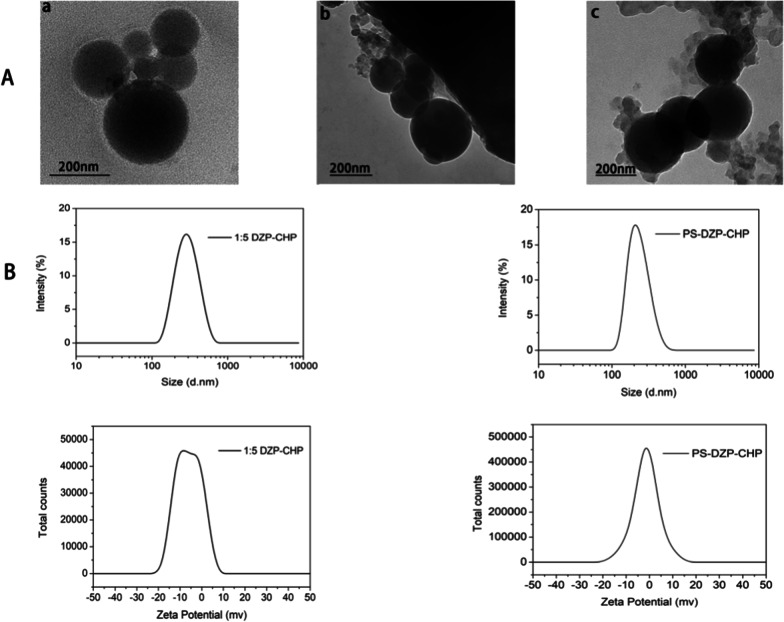
Characterization of different nanoparticles. **a** a Transmission electron microscopy photograph of CHP NPs, **b **transmission electron microscopy photograph of DZP-CHP NPs, **c **transmission electron microscopy photograph of PS-DZP-CHP NPs. B: Particle size diagram and zeta potential diagram of CHP-DZP NPs (feed ratio 1: 5) and DZP-CHP NPs modified with polysorbate 80 (feed ratio 1: 5)

The CHP NPs and DCNs prepared by dialysis showed a uniform spherical shape (Fig. 3a). According to the above study, we chose the DZP-CHP nanoparticles with a drug to nanomaterials ratio of 1:5 as the subjects of the following experiment. The DZP-CHP nanoparticles had a relatively uniform particle size of 260.7 ± 1.76 nm, and the dispersion index was 0.196 ± 0.019. Although the particle size remained relatively stable after drug loading, it sharply increased to 335.2 ± 5.46 nm after PS adsorption. The zeta potential of the DZP-CHP nanoparticles was − 0.66 ± 0.04 mV, and after coating with polysorbate 80, the zeta potential dropped to − 2.22 ± 0.86 mV (Fig. 3b).

### In Vitro Drug Release of Nanoparticles

**Fig. 4:**
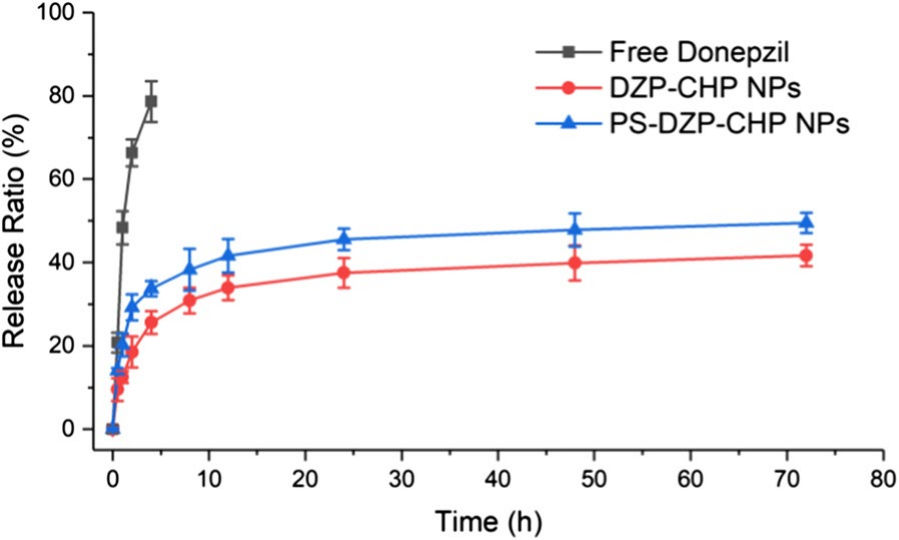
In vitro drug release curves for DZP, DZP-CHP NPs (1:5) and PS-DZP-CHP NPs

The results showed that compared with free donepezil, DZP-CHP NPs and PS-DZP-CHP NPs released DZP for 72 h with obvious controlled release effects. The early rapid release rate of drug-loaded nanoparticles may be due to the rapid dissolution and release of drug molecules, and then, the slow-down may be caused by the decrease in drug concentration, which can only be affected by dissolution and diffusion. The in vitro drug release of DZP-CHP NPs coated and uncoated with polysorbate 80 was then studied. The reason for the slower release of PS-DZP-CHP NPs may be due to strong adsorption of polysorbate 80 to small hydrophobic molecular drugs (Fig. 4).

### Nanoparticle Brain Targeting Effect

#### Observation of Brain Targeting Using Live Fluorescence Imaging Technology

**Fig. 5:**
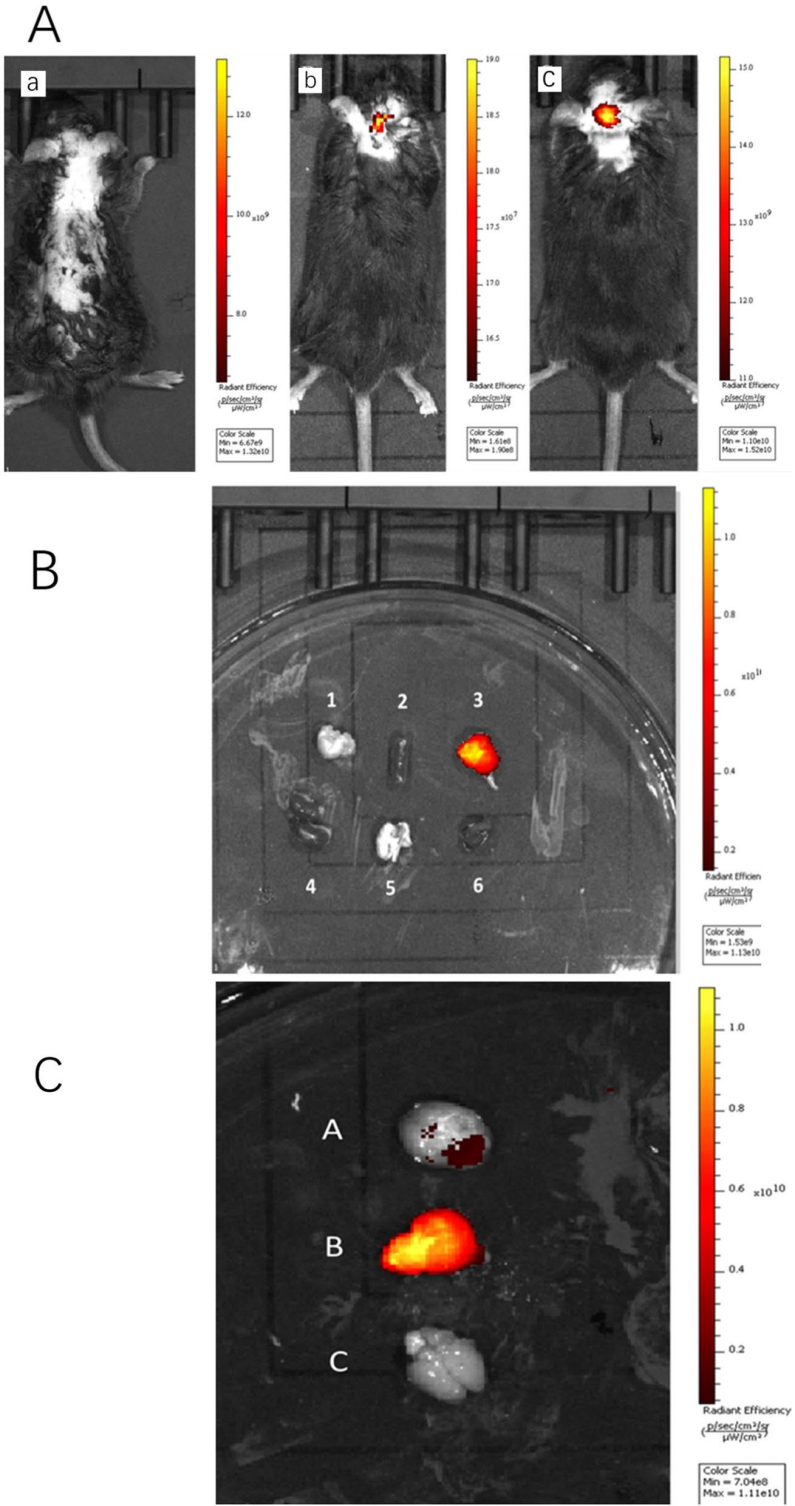
In vivo fluorescence images after different solutions were injected via the tail vein. **a** Fluorescence images of whole animals injected via the tail vein with 200 μg/ml DZP-CHP or PS-DZP-CHP nanoparticles loaded with ICG as a stain. a Free ICG solution (fluorescence intensity × 10^9^), **b** ICG-DZP-CHP nanoparticles (fluorescence intensity × 10^7^), **c** ICG-DZP-CHP nanoparticles modified by PS (fluorescence intensity × 109). ** b** Fluorescence images of various organs after injection of DZP-CHP nanoparticles modified by PS via the tail vein. **c** Fluorescence images of the brain after dissection. **d** Brain of mice injected with ICG-PS-DZP-CHP nanoparticles, **e** brain of mice injected with ICG-DZP-CHP nanoparticles modified with PS, **f** brain of mice injected with free ICG

The brains of mice injected with free ICG showed no fluorescence, but the brains injected with nanoparticles emulsified with PS presented stronger fluorescence in the brain than those injected with nonemulsified nanoparticles because both nanoparticles were able to reach the brain after injection through the tail vein (Fig. 5a). To verify this, we dissected the mice 30 min after intravenous injection of ICG-DZP-CHP and PS-ICG-DZP-CHP solutions, removed all the organs needed for the study, and then performed fluorescence imaging. PS-ICG-DZP-CHP nanoparticles presented strong fluorescence in the brain, but none was observed in other organs (Fig. 5b). Images showed that nanoparticles modified with PS presented the strongest fluorescence in brain tissue, while those not modified showed weak fluorescence. No fluorescence was observed in the brain tissue of mice injected with free ICG (Fig. 5c).

#### Tissue Distribution of Nanoparticles in Mice

**Fig. 6:**
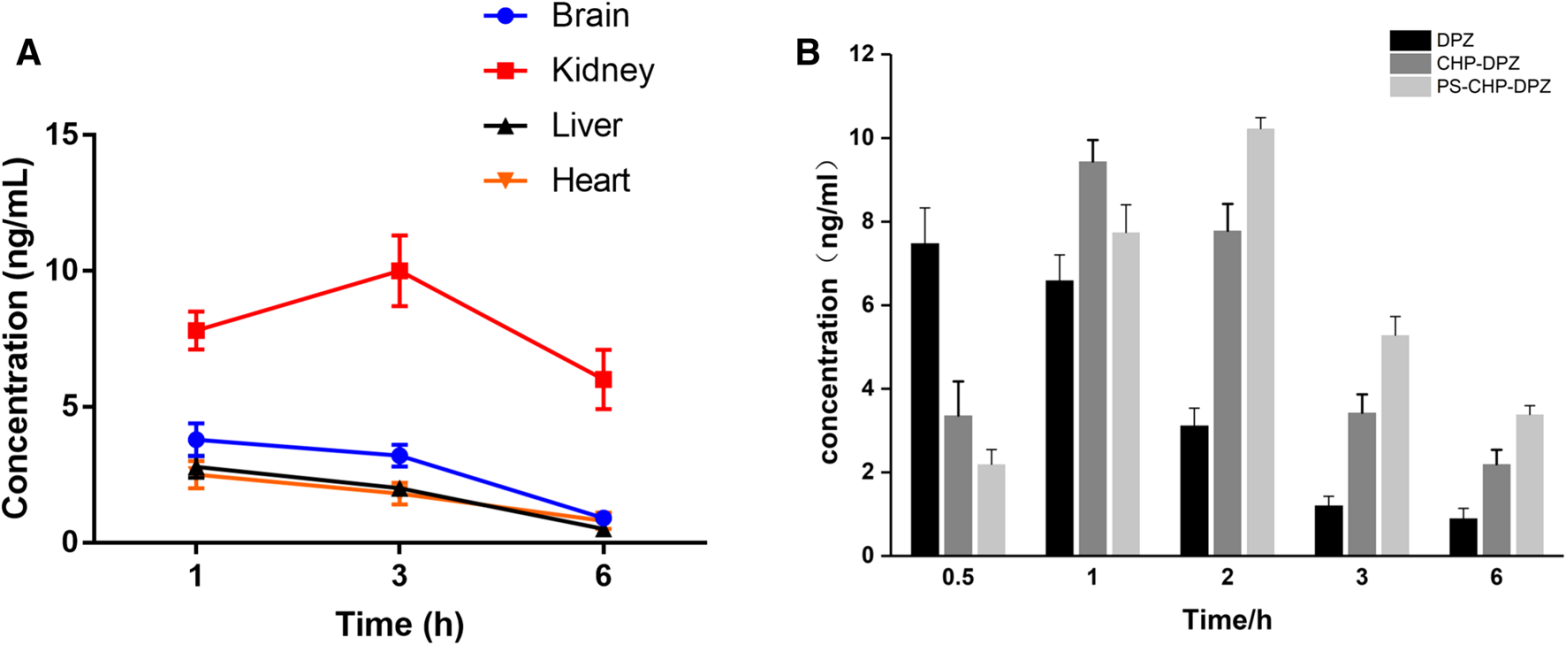
**a** The concentration of donepezil in the brain, heart, liver and kidney at different times. **b** The concentrations of free DZP, DZP-CHP nanosolution and DZP-CHP nanosolution modified with PS in brain tissue at different times

Donepezil was distributed in various tissues and mainly in the brain after injection of PS-DZP-CHP nanoparticles. Because it is metabolized through the kidney, the concentration of donepezil is very high in the kidney at certain times (Fig. 6a). In the brain, the concentration of free donepezil reached a peak in a very short time and then decreased rapidly. However, the concentration of donepezil nanoparticles reached a peak much more slowly and then decreased, especially nanoparticles modified with PS, which indicates a sustained-release effect with a delayed peak and prolonged retention time. Obviously, the nanoparticles improved the bioavailability of the drug (Fig. 6b).

#### MST Results

**Fig. 7:**
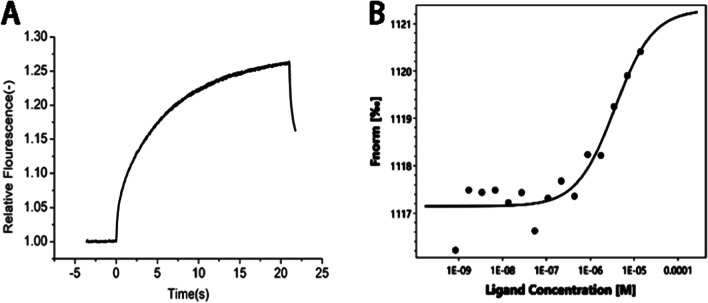
**a** Raw MST data. The fluorescently labeled molecules were observed for 5 s. At this time, the infrared laser was turned on, and a small part of the capillary tube was heated to 2–5 °C. The molecules migrated along a thermal gradient, resulting in changes in fluorescence intensity. When the infrared laser is turned off, the molecules diffuse along the concentration gradient. **b** The binding curve is generated by the difference between the initial fluorescence intensity and the intensity in the presence of heat, and the curve conforms to the standard 1:1 binding model

MST results showed that the thermal surge changed regularly with increasing ligand concentration, and the K_D_ value was 3.63 μM, indicating that the ligand effectively binds to the target protein, which verifies that PS can bind to Apo E and is relatively stable. After surface modification with PS, CHP nanoparticles can promote adsorption of Apo E, theoretically confirming that the nanoparticles we designed can specifically target brain tissue because the nanoparticles adsorbed Apo E, which may mediate passage through the blood–brain barrier (Fig. 7).

### Establishment of a Nerve Injury Model Induced by Aβ_25–35_

**Fig. 8:**
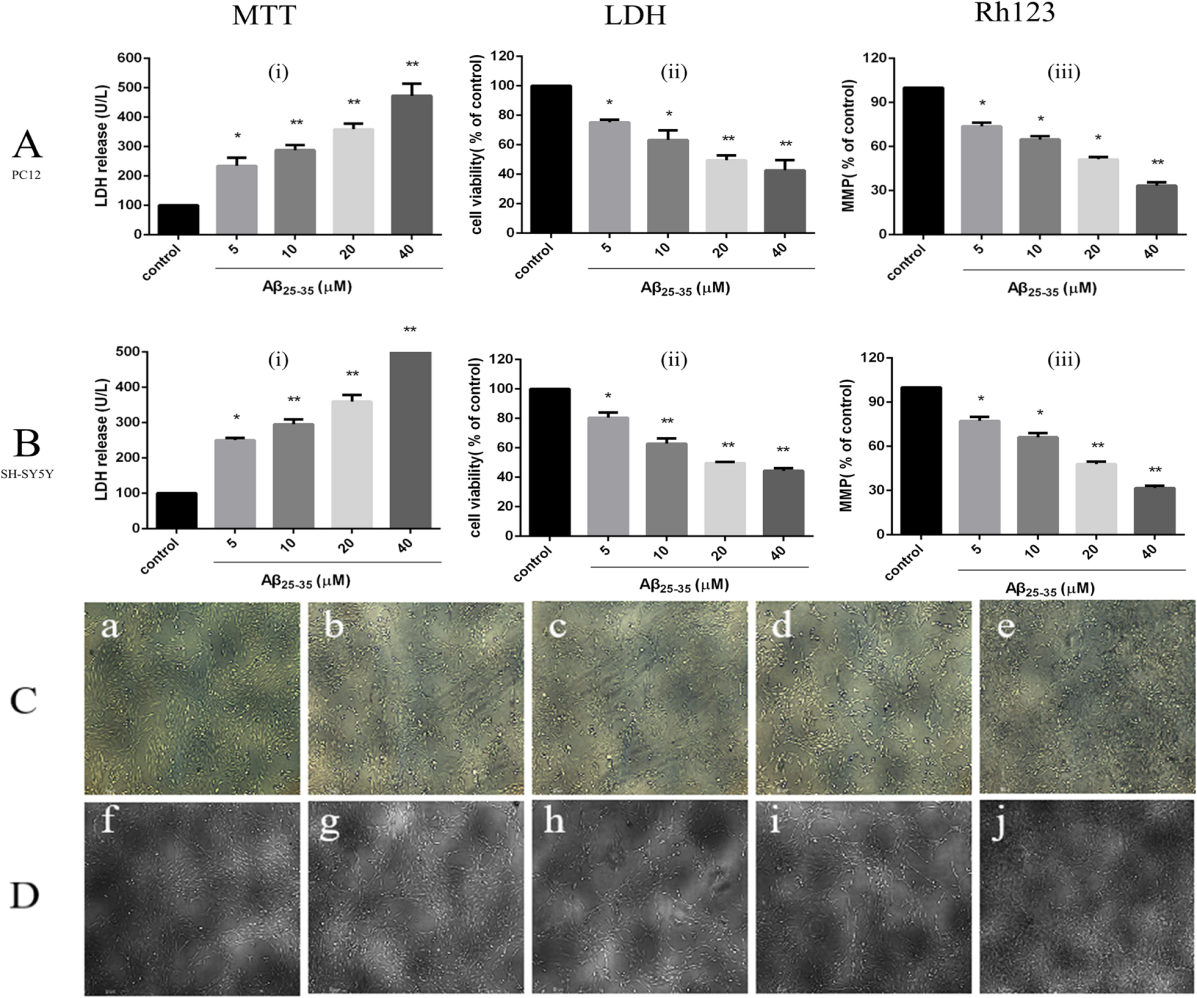
Effects of different concentrations of Aβ_25–35_ on injury to PC12 and SH-SY5Y cells. **a**, **b** The effect of different concentrations of Aβ_25–35_ on the cell survival rate, LDH activity and cell mitochondrial membrane potential in PC12 cells and SH-SY5Y cells (*P < 0.05, ** *P* < 0.01 vs control group). **c** The effect of different concentrations of Aβ_25**–**35_ on damage to the morphology of PC12 cells: **a **control group, **b **Aβ_25**–**35_ (5 μM) injury group, **c **Aβ_25**–**35_ (10 μM) injury group, **d **Aβ_25**–**35_ (20 μM) injury group, **e **Aβ_25**–**35_ (40 μM) injury group. D: the effect of different concentrations of Aβ_25**–**35_ on injury to the morphology of SH-SY5Y cells: f—control group, **g **Aβ_25**–**35_ (5 μM) injury group, h—Aβ_25**–**35_ (10 μM) injury group, i—Aβ_25**–**35_ (20 μM) injury group, j—Aβ_25**–**35_ (40 μM) injury group

MTT tests were used to detect the effect of different concentrations of Aβ25-35 on the activity of PC12 and SH-SY5Y cells [Fig. 8a (i), Fig. 8b (i)], and the results showed that with increasing Aβ_25–35_ concentration, PC12 and SH-SY5Y cell proliferation activity gradually decreased compared with that in the normal control group. When PC12 and SH-SY5Y cells were treated with 20 μM Aβ_25–35_, PC12 cell activity decreased to 49.5 ± 3.3% that observed in the control group (*P* < 0.01), and SH-SY5Y cell activity decreased to 49.7 ± 0.8% (*P* < 0.01). An LDH kit was used to detect the effect of different concentrations of Aβ_25–35_ on LDH activity in both cell supernatants. Colorimetric tests showed that activity in both supernatants increased gradually with increasing Aβ25–35 concentration. After treatment of the cells with 20 μM Aβ_25–35_, PC12 cell LDH release increased to 359.3 ± 18.3% that in the control group (*P* < 0.01), and SH-SY5Y cell LDH release increased to 360.0 ± 18.2% (*P* < 0.01). Rhodamine 123 staining was used to detect the effect of different concentrations of Aβ_25**–**35_ on the mitochondrial membrane potential in both cell lines [Fig. 8a (ii), b (ii)]. The test showed that the mitochondrial membrane potential in both cell lines decreased gradually with increasing Aβ_25**–**35_ concentration (5, 10, 20, 40 μmol/L). Treatment of the cells with 20 μM Aβ_25–35_ decreased the PC12 cell mitochondrial membrane potential to 51.3 ± 1.6% that in the control group (*P* < 0.01); for SH-SY5Y cells, the MMP decreased to 47.9 ± 1.7% that in the control group (*P* < 0.01).

Inverted fluorescence microscopy was used to observe morphological changes of PC12 and SH-SY5Y cells injured by different concentrations of Aβ_25**–**35_ (Fig. 8c, d). The PC12 and SH-SY5Y cells in the control group had higher density, fusiform shapes, fuller cell bodies and longer protrusions. As the concentration of Aβ_25**–**35_ increased (5, 10, 20, 40 μmol/L), the number of cells in both cell lines gradually decreased, the cell bodies shrank slightly, and the protrusions began to shrink sharply. When the concentration of Aβ_25**–**35_ was increased to 40 μmol/L, the protrusions broke significantly, most of the cells contracted sharply, their shape became irregular, and some cells detached and became suspended in the solution.

Therefore, we treated PC12 and SH-SY5Y cells with 20 μM Aβ25-35 for 24 h to establish a nerve injury model.

### Neuroprotective Effect of Drug-Loaded Nanoparticles (DZP-CHP)

**Fig. 9:**
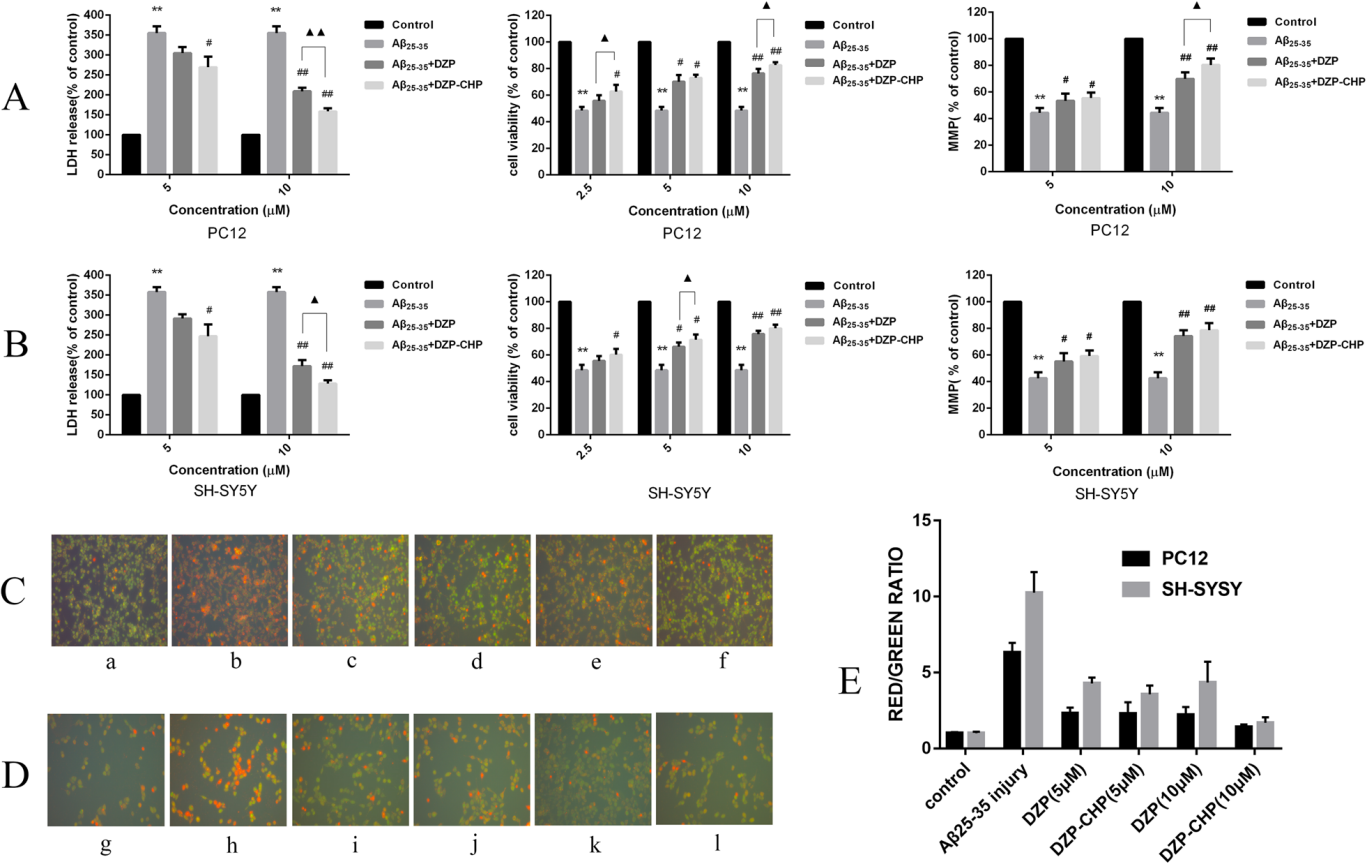
Effect of DZP-CHP on Aβ25-35-injured PC12 and SH-SY5Y cells. **a** and **b** The effects of DZP-CHP on the cell survival rate, LDH activity, and mitochondrial membrane potential of PC12 and SH-SY5Y cells injured by Aβ25-35 (#*P* < 0.05, ## *P* < 0.01 vs Aβ25-35 group; ▲ *P* < 0.05 vs DZP group). **c** The effect of DZP-CHP on the apoptosis morphology of PC12 cells injured by Aβ25-35; a—control group, b—Aβ25-35 injury group, c—DZP (5 µM), d—DZP -CHP (5 µM), e—DZP (10 µM), f—DZP-CHP (10 µM). D: the effect of DZP-CHP on the apoptosis morphology of SH-SY5Y cells injured by Aβ25-35; g—control group, h—Aβ25-35 injury group, i—DZP (5 µM), j—DZP-CHP (5 µM), k—DZP (10 µM), l—DZP-CHP (10 µM). E: red/green fluorescence ratio

MTT assays were used to detect the activity of different concentrations of DZP and DZP-CHP (2.5 μM, 5 μM, 10 μM) in PC12 and SH-SY5Y cells [Fig. 9a (i), b(i)]. Tests showed that treatment of PC12 cells with 20 μM Aβ25-35 alone resulted in a significant reduction in cell viability to 48.4 ± 2.8% that in the control group (*P* < 0.01). However, after pretreatment with DZP and DZP-CHP (2.5 μM, 5 μM, 10 μM) solutions, the viability of PC12 cells increased significantly. The viability of PC12 cells in the DZP-CHP group was higher than that in the DZP group (*P* < 0.05). Similarly, treatment of SH-SY5Y cells with 20 μM Aβ_25**–**35_ alone resulted in a significant reduction in cell viability to 48.5 ± 4.0% that in the control group (*P* < 0.01), while after pretreatment with DZP and DZP-CHP solution (2.5 μM, 5 μM, 10 μM, respectively), the viability of SH-SY5Y cells increased significantly. The viability of SH-SY5Y cells in the DZP-CHP group was higher than that in the DZP group (*P* < 0.05).

LDH kits were used to detect the effects of different concentrations of DZP and DZP-CHP (5 μM and 10 μM) on the release of LDH from PC12 and SH-SY5Y cells into the culture medium [Fig. 9a (ii), b (ii)]. Colorimetric measurements showed that the release of LDH from PC12 cells that were exposed to 20 μM Aβ25-35 alone increased significantly by 355.1 ± 16.6% (*P* < 0.01). In the presence of DZP and DZP-CHP (5 μM and 10 μM), LDH release from PC12 cells dropped significantly. The effect in the DZP-CHP group was higher than that in the DZP group (*P* < 0.01). Similarly, the release of LDH from SH-SY5Y cells exposed to 20 μM Aβ25-35 increased significantly to 357.8 ± 12.5% (*P* < 0.01). However, after pretreatment with DZP and DZP-CHP (5 μM and 10 μM), LDH release from SH-SY5Y cells decreased significantly. The effect in the DZP-CHP group was higher than that in the DZP group (*P* < 0.05).

According to previous reports, depolarization of the MMP leads to loss of Rh123 from mitochondria, which in turn leads to a decline in intracellular fluorescence. Therefore, to characterize changes in the mitochondrial membrane potential in PC12 and SH-SY5Y cells treated with Aβ25-35, DZP, and DZP-CHP (5 μM, 10 μM), rhodamine 123 was used for detection [Fig. 9a (iii), b (iii)]. The results showed that the fluorescence intensity of rhodamine 123 decreased significantly to 44.3 ± 3.8% (*P* < 0.01) after incubation of PC12 cells with 20 μM Aβ25-35 for 24 h. However, pretreatment with DZP and DZP-CHP (5 μM, 10 μM) solutions resulted in a significant increase in fluorescence intensity in a dose-dependent manner, and the effect in the DZP-CHP group was higher than that in the DZP group (*P* < 0.05). Similarly, after treatment of SH-SY5Y cells with 20 μM Aβ25-35 for 24 h, the fluorescence intensity of rhodamine 123 significantly decreased to 42.5 ± 4.6% (*P* < 0.01). However, after pretreatment with DZP and DZP-CHP (5 μM, 10 μM), the fluorescence intensity increased significantly, and the effect in the DZP-CHP group was higher than that in the DZP group.

An AO-EB double staining kit was used to detect morphological changes of PC12 and SH-SY5Y cells treated with different concentrations of DZP and DZP-CHP (5 μM and 10 μM) (Fig. 9c, d). After AO-EB double staining, the nuclei of living cells presented green fluorescence under a fluorescence, and the fluorescence of apoptotic cells was orange-red; the higher the degree of apoptosis is, the brighter the fluorescence. Compared with the untreated control group, PC12 and SH-SY5Y cells treated with Aβ25-35 alone showed typical apoptotic characteristics, such as highly condensed and broken nuclei and obvious cell injury. However, pretreatment with DZP and DZP-CHP solution (5 μM and 10 μM) significantly inhibited cell damage and improved cell morphology.

## Discussion

Although nanoparticles have been shown to be an effective delivery medium for nervous system diseases, the complexity of their structure and performance makes it challenging to detect and evaluate their physical–chemical properties and biological safety [34–36]. Therefore, after nanodrugs are designed, experiments to verify the safety and effectiveness of the nanomaterials at the cell and animal levels are extremely necessary. In the early laboratory stage, PS-DZP-CHP nanoparticles were successfully synthesized, and the optimal dosing ratio of the drug to CHP was set at 1:5. Due to the stable adsorption of PS to apolipoproteins ApoB and ApoE, brain targeting of nanoparticles can be achieved by permeation through the BBB [37]. The expected goal is that nanoparticles begin to decompose after reaching the brain; DZP is released and increases the concentration of cholinesterase; CHP reduces Aβ protein deposition and improves the brain environment; and administration frequency decreases because of the sustained release from nanoparticles [38–41].

Therefore, this study conducted an in vitro drug release test to assess the sustained release effect of nanoparticles. Compared with free DZP, nanoparticles achieved local sustained release in the brain, and PS can adsorb plasma proteins and reduce the loss of nanoparticles and prolong the release time to achieve a long cycle. After injection of ICG-labeled DZP-CHP and PS-DZP-CHP nanoparticles into rats, emulsified nanoparticles showed stronger fluorescence in the brain than those not emulsified with Tween 80. After organ biopsy, fluorescence imaging of various organs revealed that only the brain presented strong fluorescence, while other organs did not, indicating that nanoparticles did not release the drug until they reached the brain, which met the expected goal. In addition, nanoparticles modified with polysorbate 80 adsorbed ApoE to the surface and simulated low-density lipoprotein to bind to lipoprotein receptors on the surface of endothelial cells and enter the brain through LDLR induction [42, 43]. Moreover, the mechanisms by which nanoparticles regulate the tight junctions between endothelial cells and the inhibition of P-glycoprotein may produce a synergistic effect on their transcellular transport into the brain parenchyma [44]. However, since polysorbate 80 is prone to produce toxic substances, which cause untoward reactions, such as hypotension, dyspnea and shock, the dosage should be strictly controlled [45, 46]. Although a large number of nanodrugs have been developed for treatment of CNS diseases, most have shown poor effects [4]. In this study, we built a brain model of AD patients to evaluate the protective effect of nanoparticles on the brain. Because the toxicity of the Aβ protein to nerve cells varies with the concentration, it is better to screen an appropriate concentration to better simulate the brain environment of AD patients. The model was treated with DZP and DZP-CHP nanosolutions in advance to investigate the protective effect of DZP-CHP against PC12 and SH-SY5Y cell damage induced by Aβ25-35. The results showed that both solutions improved the cell proliferation activity caused by Aβ25-35, LDH release declined, and the mitochondrial potential rose. The inhibitory effect of the DZP-CHP nanosolution on cell damage induced by Aβ25-35 was significantly better than that of free DZP solution, and a concentration of 10 M DZP-CHP nanosolution proved to be the best, which may be due to the optimal drug concentration approach.

Basically, this study deduced the activity process of nanoparticles in *vivo*, verifying that PS-DZP-CHP nanoparticles have strong brain targeting, a good sustained release effect and a good AD therapeutic effect, thus demonstrating that they are clinically promising nanodrugs. The difficulty of treating the brain with medication has always been a major problem for researchers. The BBB leads to difficulty in curing CNS diseases, such as Parkinson's disease, brain tumors and brain stroke [47]. PS-DZP-CHP nanoparticles can effectively pass the BBB without destroying it, and DZP can be replaced as a model drug. Loading other drugs with high fat solubility and poor dissolution in *vivo* into the hydrophobic center of the NPs can not only increase brain targeting but also improve the water solubility of the drug. In addition, the design of CHP nanoparticle solutions is simple, and the drug dosage of the hydrophobic center can be flexibly controlled to ensure the best therapeutic effect while minimizing cytotoxicity [48, 49]. In future work, we will conduct in *vivo* research on DZP-CHP nanoparticles, such as evaluation of drug metabolism and side effects. These studies supplied a new strategy for brain drug delivery, and it is advantageous to clinical application of nanodrug with AD treatment.

## Conclusion

DZP-CHP nanoparticles showed an optimal drug to nanomaterials dosing ratio of 1:5, which led to higher PS coverage and drug loading. DZP-CHP nanoparticles with PS adsorption exhibited slow release and significant brain targeting. Nanoparticle surface modification with PS can promote adsorption of Apo E and thus is vital for brain targeting. DZP-CHP nanoparticles had a protective effect on neurotoxicity and were superior to free donepezil.

**Table 1:** Characteristics of nanoparticles

Nanoparticles	Mean particle size (nm)	Polydispersity index (PDI)	Zeta potential (mV)	Percentage of drug entrapment	Percentage of drug loading
CHP	257 ± 3.05	0.169 ± 0.02	− 2.81 ± 0.27	−	–
DCN1	273 ± 3.72	0.138 ± 0.013	− 6.20 ± 0.40	42.00 ± 5.65	12.02 ± 1.90
DCN2	260.7 ± 1.76	0.196 ± 0.019	− 5.75 ± 0.64	86.54 ± 1.31	13.42 ± 2.03
DCN3	266.8 ± 4.56	0.123 ± 0.004	− 9.30 ± 0.39	59.71 ± 4.43	7.40 ± 1.72

## Data Availability

Not applicable.
